# A systematic review and meta-analysis of the association between neglected tropical diseases and malnutrition: more research needed on diseases other than intestinal parasites, leishmaniasis and leprosy

**DOI:** 10.1099/acmi.0.000800.v3

**Published:** 2024-11-13

**Authors:** Aloysius Loglo, Wilfred Aniagyei, Monika Mira Vivekanandan, Abigail Agbanyo, Evans Adu Asamoah, Richard O. Phillips, Reginald Annan, Barbara Engel, Rachel E. Simmonds

**Affiliations:** 1 Department of Microbial Science, School of Biosciences, Faculty of Health and Medical Sciences, University of Surrey, Guildford, UK; 2 Kumasi Centre for Collaborative Research in Tropical Medicine (KCCR), Kwame Nkrumah University of Science and Technology, Kumasi, Ghana; 3 Department of Biochemistry and Biotechnology, College of Sciences, Kwame Nkrumah University of Science and Technology, Kumasi, Ghana; 4 Department of Nutritional Sciences, School of Biosciences, Faculty of Health and Medical Sciences, University of Surrey, Guildford, UK

**Keywords:** meta-analysis, NTDs, nutrition, systematic review

## Abstract

**Background.** According to the World Health Organization, neglected tropical diseases (NTDs) affect over two billion people worldwide. While the links between nutrition and many diseases have become clear over recent decades, NTDs have lagged behind and the linkage with nutrition is largely unknown. We conducted this systematic review with meta-analysis to determine the current knowledge on the association between NTDs and malnutrition.

**Methodology.** PubMed, Embase, Scopus and African Journals Online databases were searched using predefined search terms. We included all original articles with a case–control design and at least one NTD. The studies had to compare nutritional parameters between infected cases and control participants. Articles that did not report original data were excluded. The quality of the studies was assessed using the Newcastle–Ottawa scale. Pooled estimates were conducted using the random effect model. The publication bias of the studies was determined by funnel plots. *Q* and *I*^2^ statistics were used to assess the heterogeneity of the studies.

**Results.** After screening 1294 articles, only 16 qualified for the systematic review and 12 for meta-analysis. These predominately had a focus on soil-transmitted helminthiasis (ascariasis, hookworm diseases and trichuriasis) and schistosomiasis, with a minority concerning leishmaniasis and leprosy. Pooled estimates showed an association between intestinal parasites and stunting in children [odds ratio (OR) = 1.38, 95% confidence interval (CI): 1.14–1.66, *I*^2^ = 0%, tau^2^ = 0]. We also identified a moderate association established between serum iron deficiency (OR = 4.67, 95% CI: 1.91–11.44, tau^2^ = 0) and intestinal parasites.

**Conclusions/significance.** Of the 20 NTDs, the links between diet and disease have been explored for only 4. There is a paucity of data from low- and middle-income countries and least-developed countries where the NTD burden is high. Therefore, more research into the role of malnutrition in NTDs other than intestinal parasites, leishmaniasis and leprosy is needed.

**Impact Statement**

In this systematic review, we aimed to discover the extent of the current research and knowledge gaps in the role of nutritional status in all 20 neglected tropical diseases (NTDs) recognized by the World Health Organization (WHO). This is important because the WHO’s roadmap for NTD eradication by 2030 aims to intensify cross-cutting approaches including public health nutrition. The main finding is that the current literature of NTD case–control studies is limited to only 4 of the 20 NTDs. It also revealed that many previous studies did not assess systemic inflammation by recommended methods, which should be performed to adjust for some micronutrient levels in biomarker analysis to avoid confounding factors. This review is therefore important to motivate NTD researchers to undertake research into the role of diet in disease aetiology, especially since many of those with NTDs live in marginalized communities where the diet of the general population is also little studied.

## Data Summary

The articles from which data for meta-analysis were extracted are all cited within the manuscript, and no other data were analysed. The authors confirm that all supporting data, code and protocols have been provided within the article or through supplementary data files.

## Introduction

The World Health Organization (WHO) recognizes a group of 20 predominantly infectious diseases based on poor funding allocated for their control and management as neglected tropical diseases (NTDs) [1,2]. NTDs affect people residing in tropical and subtropical climates, and some disproportionately affect women and children [3,4]. Globally, 2.7 billion people from 149 countries are affected by these diseases [5]. In endemic tropical countries, the poorest of poor people residing in rural remote communities are disproportionately affected [3]. Seventy-three per cent of Africa's disease burden is from communicable diseases, which account for 71% of deaths. Among these, neglected tropical diseases (NTDs) cause a fifth of the fatalities.

NTDs, if not treated early, can result in death or lifelong disability, which negatively affects the quality of life, employment status, educational progress and social well-being due to discrimination [3]. Pathogens causing NTDs are broadly grouped as helminths, protozoa, bacteria, fungi or viruses. NTDs caused by helminth infections include soil-transmitted helminthiasis [STH; ascariasis (roundworm), hookworm, trichuriasis (whipworm) and strongyloidiasis (threadworm)], schistosomiasis (bilharzia), dracunculiasis (guinea worm), echinococcosis (flatworms), foodborne trematodiases [a broad name representing liver flukes (clonorchiasis, fascioliasis and opisthorchiasis) and lung flukes (paragonimiasis)], lymphatic filariasis (elephantiasis), onchocerciasis (river blindness) and taeniasis/cysticercosis (tapeworms). Protozoan infections include Chagas disease (*Trypanosoma cruzi* infections), human African trypanosomiasis (*Trypanosoma brucei*, sleeping sickness) and leishmaniasis (Kala-azar; three distinct clinical forms: visceral, cutaneous and mucocutaneous). The NTDs caused by bacterial infections are Buruli ulcer (*Mycobacterium ulcerans* infection), leprosy (*Mycobacterium leprae* infection and Hansen’s disease), trachoma and yaws (endemic treponematoses), while fungal infections include mycetoma (Madura foot and other deep mycoses). Viral infections include dengue (breakbone fever), chikungunya and rabies (hydrophobia or lyssa). NTDs also include ectoparasitic infestations such as scabies (sarcoptic mange) and the non-infectious disease/condition snakebite envenoming [6].

Many people with NTDs are infected with intestinal parasites [7,8]. STHs are considered as a single NTD due to their similarity in diagnosis and treatment approaches. The main infectious STH species are *Ascaris lumbricoides*, *Ancylostoma duodenale*, *Necator americanus* and *Trichuris trichiura* [9]. *Strongyloides stercoralis* is also considered an STH, although it is not detected using standard diagnostic approaches. Conversely, schistosomiasis (caused by *Schistosoma guineensis*, *S. intercalatum*, *S. japonicum*, *S. mansoni* or *S. mekongi*) is detected by the same diagnostic approach but is considered a separate NTD by the WHO due to its transmission life cycle that involves freshwater snails [9]. It should be noted that another schistosome, *S. haematobium*, primarily affects the urinary system and is therefore not considered an intestinal parasite.

Most NTDs are prevalent in countries where there is widespread evidence of malnutrition, defined by the WHO as deficiency, overabundance or imbalance in an individual’s energy and/or nutrient intake [10]. Malnutrition occurs in two forms, undernutrition or overweight/obesity. Undernutrition, resulting from inadequate nutrient intake and/or infections, manifests as stunting [low height-for-age (HA)], wasting (low weight-for-height), underweight [low weight-for-age (WA)] and micronutrient deficiencies (lack of vital vitamins and minerals), which are common in low- and middle-income countries. On the other hand, overweight/obesity results from excessive energy intake or an imbalance between intake and utilization of energy.

The association between malnutrition and growth, gender, household income and age is now well established. For instance, a systematic review and meta-analysis of 39 Ethiopian studies found that stunting and wasting/thinness were prevalent (more than one-fifth of the population) [11]. However, it is important to understand the dietary patterns in a country as a whole, as well as dietary changes that are taking place in modern society and consider the differences between rural and city dwellers. A systematic review and meta-analysis of the changing dietary patterns of people residing in Ghana and Kenya between 1971 and 2018 found that the consumption of processed meat, cakes, sweets, soft drinks and juices increased as the years went by [12]. This ‘nutritional transition’ has led to a higher prevalence of overweight/obesity and malnutrition-related non-communicable diseases in more recent years [12]. Although countries such as Cape Verde, Ghana and Senegal are at the latter stages of the nutrition transition, evidence shows that there is an increased intake of dietary energy, sugars and protein with a consistent decrease in fruit and vegetable consumption. The change has been reported to be driven by people with a high socioeconomic class in the cities but not rural dwellers [13].

In 1968, the WHO first recognized the interconnected relationship between nutrition, infection and immunity [14]; i.e. that infections can impact the nutritional status of people just as diet does. These concepts have been thoroughly studied in several infectious and non-infectious diseases over the years [15]. However, we suspected that NTDs are under-represented in such analysis. Moreover, despite the existence of articles on the role of nutrition on a few NTDs, particularly intestinal parasites, there is currently no literature review that consolidates these findings from the existing primary research to consolidate knowledge on the impact of diet on NTDs. Therefore, the objective of this review is to synthesize the existing evidence to determine the associations between NTDs and malnutrition and identify research gaps.

## Methods

### Protocol development and registration

We developed and registered the study protocol in the International Prospective Register of Systematic Reviews (PROSPERO) database (CRD42021275523CRD42021275523). This review was prepared using the updated Preferred Reporting Items for Systematic Review and Meta-Analysis (PRISMA 2020) guidelines [16].

### Eligibility criteria

#### Inclusion criteria

The population, intervention, comparator and outcome framework was used to determine the different aspects of this review to help identify the relevant studies. Studies met the inclusion criteria if they were published, peer-reviewed original articles that involved a case–control design model of at least one NTD. Also, the studies must show evidence of assessing the nutritional parameters of infected human participants and controls with no age restriction.

#### Exclusion criteria

Articles were excluded if full-text versions were not available and not written in English language. Animal studies, ecological studies, abstracts and grey literature such as graduate study theses were excluded from the review. Also excluded were conference posters, abstracts from scientific correspondence and letters to editors.

### Search strategy

Before the electronic search on 15 September 2021, we conducted a preliminary search to agree on the search terms. The pre-search revealed that the NTDs mycetoma, chikungunya, echinococcosis, taeniasis, trematodiasis and snakebite did not identify a single article related to the focus of our review. As a result, we removed those diseases from the original search terms of 20 diseases but included the term ‘neglected tropical diseases’ to ensure no disease was excluded. The electronic search engines were PubMed, Science Direct, Embase, African Journals Online and Scopus. The search queries included combinations of disease terms such as ‘NTD’ OR ‘Neglected tropical diseases’ OR ‘Ascariasis’ OR ‘Trichuriasis’ OR ‘Hookworm infection’ OR ‘Schistosomiasis’ OR ‘lymphatic filariasis’ OR ‘Trachoma’ OR ‘Onchocerciasis’ OR ‘Leishmaniasis’ OR ‘Chagas disease’ OR ‘Leprosy’ OR ‘Human African trypanosomiasis’ OR ‘Dracunculiasis’ OR ‘Buruli ulcer’ OR ‘Yaws’ OR ‘Scabies’ in combination with nutritional terms such as ‘nutritional status’ OR ‘macronutrients’ OR ‘micronutrients’ OR ‘anthropometrics’ OR ‘dietary intake’. Details of the search terms and their combinations are provided in Table S1 (available in the online version of this article) (search strategy).

### Screening

All records identified were imported to Mendeley or Endnote to allow for the removal of duplicates from the search engines. Articles not published in the English language were also removed because we do not have a native translator in the review team and the team is made of researchers whose native language is English. This language restriction has been shown not to cause systematic bias, e.g. [17].

Afterwards, a second screening using titles and abstracts was performed on the remaining records. The screening processes were conducted independently by three skilled researchers (W.A., A.A. and M.M.V.) based on the keywords (exposure and outcome variables in Table S1) used in the online searches. The full-text versions of the articles that qualified from the previous screening were then screened by the same researchers based on the inclusion and exclusion criteria. Subsequently, the potentially eligible records were selected and carefully read. Disparities between the reviewers’ results were resolved via discussion with a fourth author (A.L.).

Records from the various stages of screening were saved on databases in an Excel format (Microsoft, WA, USA) and organized in Mendeley or Endnote software.

### Data extraction

A data extraction sheet was prepared using Microsoft Excel (Microsoft) spreadsheet. A draft extraction spreadsheet was first piloted and refined, and a final version was agreed upon by the four reviewers (A.L., W.A., A.A. and M.M.V.). The full text of eligible articles was obtained, and data relevant to this review were extracted.

The following information was extracted using the data extraction sheet: the name of the first author and year of publication, country of the study type of NTD(s), the sample size for cases and controls, the type of organism, type of sample obtained for biomarker assessment, tools/methods used for nutritional assessment (e.g. anthropometrics and 24-h recall), analytes quantified (nutrients and immunological markers), methods for analyte quantitation (e.g. HPLC and ELISA). Other primary outcome measures for individual studies were also noted.

### Quality assessment

The Newcastle–Ottawa scale (NOS) was used to assess the quality of the studies and determine the articles that met the inclusion criteria (Table S2). The NOS was conducted independently by two assessors (A.A. and M.M.V.) who later compared each quality scale. Two reviewers (A.L. and W.A.) conducted checks of every agreed score to conclude. The definitions of the scores are as follows: *good quality score*, 3 or 4 stars in the selection domain and 1 or 2 stars in the comparability domain and 2 or 3 stars in the outcome/exposure domain; *fair quality score*, 2 stars in the selection domain and 1 or 2 stars in the comparability domain and 2 or 3 stars in the outcome/exposure domain; and *poor quality score*, 0 or 1 star in the selection domain or 0 star in the comparability domain or 0 or 1 star in the outcome/exposure domain [18]. A scale of 4 and below was considered poor and thereby the cut-off.

### Data synthesis and analysis

The process of data collation and synthesis followed the guidelines of PRISMA 2020 [16]. R software (version 4.1.0; SC, USA) was used for the meta-analysis using the ‘Meta’ package, considering each NTD (or NTD group) separately.

The analysis of body mass index (BMI)-for-age (BAZ) and HA Z score (HAZ) and nutritional deficiencies/insufficiencies was based on the number of individuals diagnosed from the cohort of cases and controls extracted for analysis. The summary measures for HAZ, BAZ and nutritional deficiencies/insufficiencies were odds ratios (ORs) and 95% confidence interval (CI). The ORs were pooled using the Mantel–Haenszel method, and 95% CIs were estimated using the Clopper–Pearson method and restricted maximum (REM) likelihood estimator for tau^2^. The CIs of tau^2^ and tau were estimated using the Q-Profile method. All studies used for the meta-analysis for BAZ and HAZ used Centers for Disease Control and Prevention or WHO standardized criteria for classification. The majority of the studies used the WHO AnthroPlus software versions 1.0.3 and 1.0.4 for anthropometry calculation [19,20]. BAZ was used to determine thinness/wasting for children ≥10 years and adolescents. Z scores of <−2 sd were used to categorize children as thin/wasted, and an HAZ score of <−2 sd was categorized as stunted. BAZ and HAZ were considered normal when the Z scores of the children were >−2 sd.

The nutritional biomarker analysis was conducted using the mean and sd extracted from the included studies. A random effect model was used to measure the standardized mean difference (SMD) and its 95% CI. The primary measures for the nutritional biomarkers were SMD and 95% CI. The SMD was used because the method used to measure the nutritional markers was not the same for all studies included in the meta-analysis. That is, the mean differences of the biomarkers in the individual studies were divided by their respective sd to get a unified scale across all studies before pooling. With this strategy, the pooled sd was adjusted for case-versus-control differences for both scale and precision of measurement calculated as well as the sample size of individual studies. This was done with the bias-corrected SMD (Hedges *g*) measure, correcting for the effect of small sample bias and the Hartung–Knapp adjustment for the random effect model. However, there was no pooling done for markers measured in single studies. The CI estimation of the pooled SMD was based on the standard normal distribution. Nutritional deficiencies/insufficiencies were determined based on comparable cut-offs reported by the authors across the selected studies per disease. For studies considering the association between nutritional deficiencies and STH, ORs and 95% CI were used as the summary measure. Data on the number of individuals diagnosed for a specific nutrient deficiency or insufficiency were extracted for cases and control from each study and pooled using the Mantel–Haenszel method. To be eligible, studies were considered when the definition and markers used for the classification of nutritional deficiencies were uniform.

The REM likelihood was used to quantify between-study heterogeneity using the heterogeneity variance (*Ƭ*^2^). For all effect estimates (either ORs or SMD), meta-regression analysis was not performed because of the small study effect obtained with the sub-group analysis.

The publication bias was assessed using a funnel plot, while *Q* and *I*^2^ statistics was used to determine the heterogeneity of the study. Heterogeneity was defined based on the following categories: *I*^2^ <25% is low, from 25 to 50% is medium and from >50% is high (which can also be identified as significant heterogeneity).

## Results

### Search results and characterization of studies

**Fig. 1.**
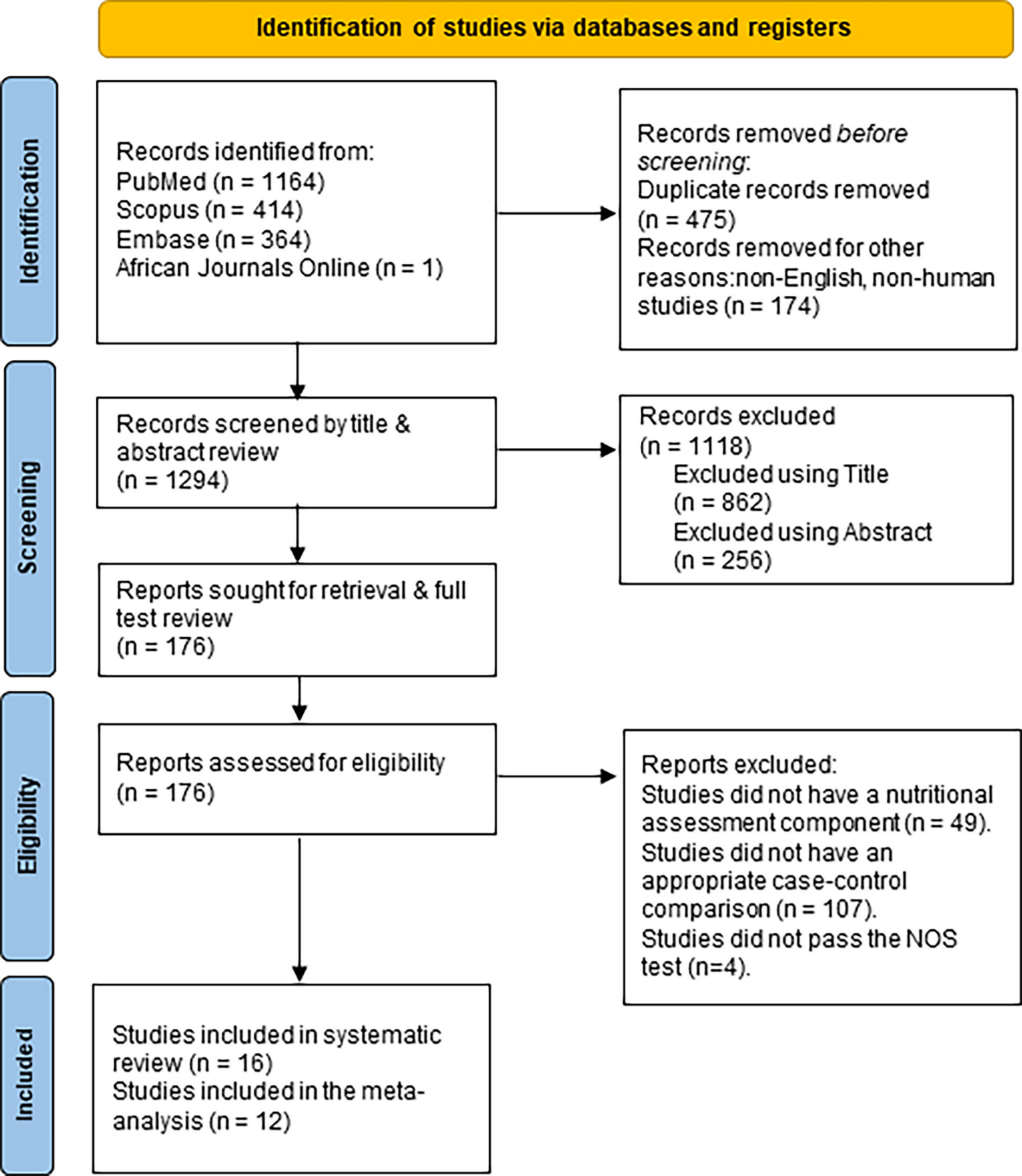
PRISMA flow diagram for the study selection process.

The search (Table S1) yielded 1943 articles with further screening leading to the removal of duplicates, articles concerning neural tube defects (which also had the acronym NTD), non-English language papers, non-human studies, publications in the form of commentaries and editorials resulting in 1294 articles. A further screening based on the inclusion criteria led to the exclusion of 1118 articles. Systematic reviews were excluded from our review because the authors would have already synthesized compiled data from original articles. In addition, the extraction parameters for this study’s meta-analysis would not be present in a review paper and thus will introduce additional heterogeneity in our pooled data. The final 176 articles were critically assessed for eligibility with a focus on studies that sought to identify associations between nutrition and NTDs with a case–control component. Forty-nine studies did not have a nutritional assessment component for the NTD(s), and 107 did not have a case–control component in the study. After a thorough screening, we further conducted the NOS quality assessment tests, which led to the removal of four more articles (Table S2). Hence, 16 full articles qualified for the systematic review. A flow chart on search strategies and articles that qualify for the review can be found in Fig. 1.

Hence, in summary, we were able to identify 16 full-text articles relating to the associations of only a small number of NTDs with diet[Table T1] (Tables1  2):

1. Intestinal parasites including STH and schistosomiasis (*n* = 9) [21,29].
2. Leishmaniasis (*n* = 5) [30,34].
3. Leprosy including one of co-infection with intestinal parasites (*n* = 2) [35,36].

**Table 1. T1:** Characteristics of included studies

First author (year)	Country	Type of NTD (organism)	Age matching, *P*-value	Age range (years)	Mean age (years)	Sample size, cases (*n*)	Sample size, controls (*n*)	Reference
Atukorala and Lanerolle (1999)	Sri Lanka	STH (*A. lumbricoides, T. trichiura, hookworm*)	na	14–18	15.5 ± 1.1§	71	545	[27]
Amare *et al.* (2013)	Ethiopia	‘Intestinal parasites’ including STH (*A. lumbricoides, hookworm, T. trichiura*) as well as *Hymenolepis nana*, *Enterobius vermicularis*, *S. stercoralis*, *Giardia lamblia*, *Entamoeba* spp*.*, *Schistosoma mansoni*	No, *P* = 0.006	ns	12.09 ± 2.5§	92	313	[21]
de Gier *et al*. (2016)	Vietnam	STH (*A. lumbricoides, T. trichiura,* hookworm)	na	6–9	7.5 ± 0.9§	409	101	[22]
Humphries *et al.* (2013)	Ghana	STH (hookworm)	Yes, *P* = 0.056	6–11	ns	109	170	[23]
Kuong *et al*. (2016)	Cambodia	STH (hookworm)	Yes, *P* = 0.654	6–15	9.8§	288	1450	[24]
Lwanga et al. (2012)	Uganda	STH (*A. lumbricoides, T. trichiura,* hookworm) and schistosomiasis (*S. mansoni*)	Yes, *P* = 0.061	6–14	10.9§	363	69	[25]
Quihui-Cota *et al.* (2010)	Mexico	STH (*T. trichiura*)	Yes, *P* = 0.92	6–10	7.6 ± 1.1§7.7 ± 1.3‡7.7 ± 1.5†	33	40	[28]
Ross *et al.* (2017)	Philippines	Schistosomiasis (*S. japonicum*)	No, *P* = 0.018	6–14	na	134	102	[26]
		STH (*A. lumbricoides, T. trichiura*, hookworm)				552	102	
Zavala *et al.* (2017)	Mexico	STH (hookworm, *A. lumbricoides*) as well as *Entamoeba coli*, *Entamoeba histolytica*, *Endolimax nana*, *Balantidium coli*, *Giardia lamblia*	na	6–10	8§	45	239	[29]
Gomes et al. (2007)	Brazil	Visceral leishmaniasis (*L. chagasi*)	Yes, ns	0–72*	17.5‡,¶16.0**,¶	107	144	[30]
					22.0††,¶24.0‡‡,¶			
Goyonlo *et al.* (2018)	Iran	Cutaneous leishmaniasis (*L. tropica*)	Yes, *P* = 0.215	ns	21.3 ± 17.6§	149	71 HHC	[34]
Kahvaz *et al.* (2020)	Iran	Cutaneous leishmaniasis (*L. major* and *L. tropica*)	Yes, *P* > 0.05	ns	31.98 ± 6.84†31.28 ± 7.40‡	80	80	[31]
Kocyigit *et al.* (2002)	Turkey	Cutaneous leishmaniasis	Yes, *P* > 0.05	9–34† 10–32‡	27.3 ± 3.8†28.4± 4.1‡	28	22	[32]
Lal *et al.* (2013)	India	Visceral leishmaniasis (*L. donovani*)	Yes, *P* = 0.382	ns	28.64†^(acute)^23.68†^(chronic)^23.05‡	44	22	[33]
Dennison *et al.* (2021)	Brazil	Leprosy (*Mycobacterium leprae*) co-infection with schistosomiasis (*S. mansoni*) or STH (*A. lumbricoides*)	† vs. NCC: yes, *P* = 0.65;† vs. HC: no, *P* = 0.005	≥3	40.0 ± 20.5§43.8 ± 20.5†35.0 ± 19.8‡	79	96 HHC, 81 NCC	[36]
de Oliveira *et al.* (2020)	Brazil	Leprosy (*Mycobacterium leprae*)	na	≥18	49.21 ± 12.35†30.3 ± 10.5‡32 ± 12.15§§	34	18 HC, 25 HHC	[35]

*Months.

†Case.

‡Control.

§All.

¶Median.

**Asymptomatic.

††Oligosymptomatic.

‡‡Active VL.

§§Household contacts.

HChealthy controlsHHChousehold contactsnanot availableNCCnon-contact controlsnsnot seenSTHsoil-transmitted helminth

**Table 2.** Methodological approaches and outcomes

First author (year)	Method (nutritional assessment)		Outcome		Reference
	Sample taken	Tool	Analytes	Main finding	
Atukorala and Lanerolle (1999)	Stool and blood samples	Anthropometry, complete blood count, ELISA	Hb and serum vitamin A	A negative association was established between serum vitamin A concentration and worm burden in subjects with moderate or heavy infection of *Trichuris* (*r*^2^ = 0.49, *F* = 8.6, *n* = 11)	[27]
Amare *et al*. (2013)	Blood samples	Anthropometry, ELISA	BMI, HAZ, WAZ, IgE	No significant association between the prevalence of allergy in this population and their nutritional status and parasite infection (*Z* = −0.198, *P* > 0.8)	[21]
de Gier *et al.* (2016)	Blood and urine samples	ELISA, HPLC, spectrophotometry	Vitamin A, Zn, ferritin, CRP, urinary iodine	A significant association was established between Ascaris infection and lower plasma retinol (aB = −0.10 mM l^−1^, 95% CI: – 0.17, –0.02.	[22]
Humphries *et al.* (2013)	Blood samples	Anthropometry, 24-h recall	BAZ, HAZ, WAZ, household hunger, household food security, animal source food, IgG	No association between anthropometric measures and hookworm infection; malaria was more common in children with hookworm infection (*P* = 0.026)	[23]
Kuong *et al.* (2016)	Blood samples	Anthropometry, ELISA, flame atomic absorption spectrophotometry	BAZ, HAZ, WAZ, Hb, RBP, ferritin, transferrin receptor, vitamin A, Fe, Zn, CRP, AGP	A negative association between body iron and increased intensity of hookworm infection (*R* = 0.22, *P* < 0.001); raw cognitive test score was significantly lower in hookworm-infected patients (*P* < 0.05)A negative correlation between body iron levels and increased intensity of hookworm infection (*R* = 0.22, *P* < 0.001)	[24]
Lwanga *et al.* (2012)	nd	Anthropometry	BAZ, HAZ, MUAC	Incidences of stunting (*P* = 0.2), underweight (*P* = 0.2) and MAM (*P* = 0.7) were attributable to helminth infection	[25]
Quihui-Cota *et al.* (2010)	Blood samples, stool	Anthropometry, 24-h recall, complete blood count	HA, WA, HW, Hb, haematocrit, RBC, MCV MCH, MCHC, serum Fe, TIBC, TS, ferritin	Lower concentrations of haemoglobin, haematocrit and serum iron in children infected with *Trichuris trichiura* (*P* < 0.05); significantly higher HAZ in the *Trichuris*-free group than in the *Trichuris*-infected group (*P* = 0.02)	[28]
Ross *et al.* (2017)	nd	Anthropometry, 24-h recall, food composition tables	BAZ, HAZ, energy, protein, total fat, carbohydrate, water, thiamine, riboflavin, niacin, vitamin C	A decreasing trend between infection intensity and the mean values of HAZ and BAZ was identified for *T. trichiura* or hookworm infectionsChildren with light hookworm infections had lower mean HAZ Z scores (–2.14 versus –1.92, *P* = 0.014) and BAZ Z scores (–1.61 versus –1.37, *P* = 0.013)	[26]
Zavala *et al*. (2017)	nd	24-h recall, FFQ	Energy, carbohydrates, protein, fat, fibre, fruits, vegetables, cereals, legumes, dairy, meat, fat, sugar, beverages	*A. lumbricoides* infection was associated with lower intake of energy (*P* < 0.01), carbohydrates (*P* < 0.01), fibre (*P* < 0.05), fruits (*P* < 0.01), cereals (*P* < 0.05), legumes (*P* < 0.05), dairy (*P* < 0.01), meat (*P* < 0.05) and sugar (*P* < 0.01)	[29]
Gomes *et al.* (2007)	Blood samples	Anthropometry, radioimmunoassay, ELISA, immunoturbidimetric test	WAZ, HAZ, WHZ, serum albumin, ferritin, GH/GF-1	A positive correlation was established between the endocrine data and the nutritional indicators irrespective of the clinical group; a statistically significant difference between HAZ and WAZ (*r* = 0.656) and albumin serum concentrations (*r* = 0.427) was seen	[30]
Goyonlo *et al.* (2018)	nd	Semi-quantitative FFQ	Vitamin A, energy, fat, protein, fibre, vitamin E and K	Macronutrient and micronutrient intake, especially vitamin A deficiency, was associated with chronic clinical disease progression	[34]
Kahvaz *et al*. (2020)	Blood samples	Flame atomic absorption spectrometer (FAAS) auto-analyser	Se, Zn, Cu, Fe	Those infected with CL presented with significantly lower serum Zn, Fe, Se and zinc/Cu ratios compared to controls (*P* < 0.001); serum levels of Cu in the CL-positive group were significantly higher than in the controls (*P* < 0.001)	[31]
Kocyigit *et al.* (2002)	Lesion smears, whole blood (after overnight fasting)	Atomic absorption spectrometer, colorimetry, chemiluminescence	Plasma Se, Cu and Zn, Fe, albumin, IL-1β, IL-2r, IL-6, IL-8, TNF-α	Positive correlations were established between selenium and IL-2r (*r* = 0.41, *P* = 0.039), copper and TNF-α (*r* = 0.34, *P* =0.044) and copper and IL-1β (*r* = 0.65, *P* = 0.006) in patients with CL	[32]
Lal *et al.* (2013)	Blood samples	Anthropometry, colorimetry	Weight, Cu, Zn, Fe, Ca, Mg	Lower levels of Zn (*P* = 0.007) and higher levels of Mg (*P* = 0.002) could be associated with chronicity in patients with visceral leishmaniasis	[33]
Dennison *et al*. (2021)	Blood samples	Complete blood count (NS for vitamins D and A)	Serum ferritin, vitamin D, vitamin A	Vitamin D deficiency was more common in leprosy cases compared to controls (aOR: 4.66, 95% CI: 1.42–15.33) (*P* < 0.05)	[36]
de Oliveira *et al.* (2020)	Blood samples	HPLC, ELISA	Vitamin D, cathelicidin	Vitamin D status and cathelicidin levels are strongly correlated during multidrug therapy for leprosy (at baseline *r* = 0.86; after 6M of MDT *r* = 0.79)	[35]

CacalciumCucopperFeironFFQfood frequency questionnaireGH/IGF-1growth hormone-insulin-like growth factor-1 axisHbhaemoglobinHWheight-for-weightIGFBP3IGF binding-protein 3IL-6interleukin 6IL-8interleukin 8IL-2rinterleukin 2 receptorIL-1βinterleukin 1 betaKpotassiumMAMmoderate acute malnutrition MCHmean corpuscular heamoglobinMCHCmean corpuscular haemoglobin concentrationMCVmean corpuscular volumeMgmagnesiumMUACmid-upper arm circumferenceNSnot seenRBCred blood cellRBPretinol-binding proteinSeseleniumTIBCtotal iron binding capacityTNF-αtumour necrosis factor alphaTStransferrin saturationWAZweight-for-age Z scoreWHZweight-for-height Z scoreZnzinc

Intestinal parasites are grouped together in this systematic review because the diagnostic technique used identifies the NTDs schistosomiasis and STH, as well as other parasitic infections, and the analyses performed in the different papers took a range of approaches to either further separate these or not. We refer to these as ‘intestinal parasites’ to avoid any confusion.

### Characteristics of eligible studies

Tables1  2 show a summary of articles that qualified for this review after the NOS quality assessment. There was no restriction on publication dates, and the studies were published between 1999 and 2021. Out of the 16 studies, 8 (50%) were conducted in Asia (Philippines [26], Vietnam [22], Cambodia [24], India [33], Sri Lanka [27], Turkey [32] and Iran [31,34]) followed by 5 (31%) in the Americas (Mexico [28,29] and Brazil [30,35, 36] and then 3 (19%) in Africa (Ethiopia [21], Ghana [23] and Uganda [25]). All ten studies of intestinal parasites (including a leprosy-intestinal parasite study) used the WHO-recommended Kato–Katz method for diagnosis. Three studies supplemented this with further analysis: PCR for hookworm [23], the Faust method [28] or the Hoffman–Pons–Janer method [36]. The two Leprosy studies diagnosed their cases first by expert dermatologists and by histopathology, ML-Flow test and bacilloscopic index [35,36]. The five studies into leishmaniasis diagnosed the disease by obtaining splenic aspirates for Giemsa stains [30,34].

Anthropometric measures involved the assessment of body weight and height to allow for the computation of several parameters, allowing the grouping of participants as normal weight, underweight, stunted, wasted, overweight or obese as recommended by the WHO [37]. Approximately half of the studies used these measures, and the majority (nine) used weight-for-age Z score, BAZ and HAZ, where the participants were children. Single studies additionally used mid-upper arm circumference (MUAC) [25] or alternatively used HA, weight-for-age and height-for-weight [28], respectively.

The tools used for dietary intake for these studies were predominantly either a food frequency questionnaire (FFQ) or 24-h recall or both. Five studies employed these self-reported recall-based approaches [23,26, 28, 29, 34]. The 24-h recall was conducted three times [26,28, 29] except for one study, which conducted the interview twice with a focus on iron status [28]. The FFQ was administered in two studies [29,34], and a third study used the information gathered to determine the dietary diversity, household food insecurity and consumption of animal sources of food [23].

Biochemical markers were determined in 11 studies (69%) with a wide variety of assays, markers and types of samples collected including serum and plasma [21,22, 24, 27, 28, 30,33, 35, 36]. Additional urine samples were also obtained in one study to quantify iodine [22]. All studies used ELISA for biomarker assessment, whereas others used an automated chemistry analyser [33], reverse-phase HPLC and/or flame atomic absorption spectrophotometer. Micronutrients were the most quantified analytes (eight studies), while immunological markers were mostly inflammation markers including C-reactive protein (CRP) and α1-acid glycoprotein (AGP) (seven studies) and (antigen specific) immunoglobulins (three studies).

### Key findings

The 16 eligible articles were grouped into 3 main diseases, namely, intestinal parasites (including 2 NTDs), leprosy and leishmaniasis. The majority of the studies concerned intestinal parasites identified within stool samples, which include STH (specifically infections with *A. lumbricoides*, *T. trichiura* and hookworm) and schistosomiasis (including *S. mansoni* and *S. japonicum*) [21,24,26, 28]. Two articles additionally identified other parasitic infections that were not strictly NTDs but are associated with poor hygiene [24,29]. Some articles went on to focus on a single infection (hookworm [23], trichuriasis [28] or *A. lumbricoides* [29]), multiple infections (all intestinal parasites, STH or schistosomiasis [26]) or compare those with or without any intestinal parasite [21].

Overall, these studies showed that micronutrient inadequacy was present in people affected by intestinal parasites, leprosy and leishmaniasis. Stunting and weight loss were associated with the presence of intestinal parasites, but not observed in leprosy and leishmaniasis. Iron deficiency was significantly more prevalent among those infected with intestinal parasites, and lower intake of energy, carbohydrates, fibre, fruits, cereals, legumes, dairy, meat and sugar was common in people infected with *A. lumbricoides*.

Meta-analysis was possible for 12 studies where we established an association between intestinal parasites and stunting. Some nutrient biomarkers were measured in plasma across all the diseases, with haemoglobin, ferritin, iron, vitamin A and zinc being the most commonly measured markers. However, vitamin D and cathelicidin were only assessed for leprosy. Vitamin D deficiency was common among the leprosy cases, while cathelicidin was strongly correlated with multiple drug resistance.

In the meta-analysis, low levels of zinc, iron and selenium were associated with leishmaniasis. Interestingly, only 2 articles (both on the topic of intestinal parasites) out of the 16 measured inflammatory markers.

Overall, macronutrient and micronutrient intake did not seem to show a significant difference between cases and controls, except for leishmaniasis where vitamin A deficiency (determined by intake) was associated with disease progression. When assessed, gender did not show a difference in food intake in children affected with schistosomiasis, but no gender comparison was made for the other diseases.

### Summary of findings

#### Anthropometry

Anthropometry was considered in cases vs. controls in 9 of the 16 studies overall: 7 concerning intestinal parasites and 2 concerning visceral leishmaniasis and none concerning leprosy (Table 2). Among studies of intestinal parasites, all these concerned children under the age of 18 years (Table 1). While most of these studies’ measures of BMI, BAZ nor HAZ did not show any significant difference between cases and controls [21,23,25, 27, 29], a study of trichuriasis (without other STH family organisms) reported the HAZ of trichuriasis-free controls to be significantly higher than that of the infected cases [28]. Moreover, a decreasing trend was established between infection intensity and the mean values of the HAZ and BAZ identified in cases of *T. trichiura* or hookworm infection compared to the uninfected cases [26].

### The extent of stunting and weight loss

The prevalence of stunting varied based on the sample size and type of NTD. In the studies of intestinal parasites, the overall prevalence of stunting was reported to range between 2 and 49.2% in five studies [21,24,26, 28]. Furthermore, the prevalence of thinness/wasting was 8.9% [21] and 27.8% [26] in two of these studies. Stunting, underweight and thinness/wasting were seen in participants not infected with intestinal parasites although this was not statistically different from the cases. Lower HAZ, BAZ and MUAC were associated with infection with intestinal parasites [25]. The prevalence of stunting was not reported for leprosy but for leishmaniasis; conducting multiple discriminant analyses revealed albumin concentration and HAZ score as predictors of active and oligosymptomatic visceral leishmaniasis [30].

### Food intake and extent of reduced intakes of nutrients

Of the 16 articles, food intake was determined in only 5, and 4 of these focused on intestinal parasites. Overall, these studies reported no difference in food consumption/nutrient intake between cases and controls. One study reported that the consumption of grains, vegetables, root/tubers and fish by those with hookworm was on (at least) a weekly basis with more than 85% of the study cohort consuming the same food groups the previous day [23]. Energy and macronutrient intake or servings per day of food groups between boys and girls were similar regardless of infection with a single or more parasites [29]. Also, in schistosomiasis, no differences were identified for nutrition indicators in infected or uninfected children except for vitamin C intake [26]. In the sole nutritional intake study on chronic cutaneous leishmaniasis, this reported food intake that had lower energy, fibre, potassium and vitamin A in cases vs. controls [34].

### Blood markers

In studies on intestinal parasites, 16 biomarkers in total were analysed over 7 studies. However, seven of these (haematocrit, red blood cell, mean corpuscular volume, mean corpuscular haemoglobin, mean corpuscular haemoglobin concentration, total iron-binding capacity and transferrin saturation) were only measured in one study [28]. The most quantified biomarkers across the studies were haemoglobin [24,27, 28] and vitamin A [22,24, 27]. Serum concentrations of zinc [22,24], ferritin [22,28] and iron [24,28] were biomarkers determined in two studies each. Total serum IgE was quantified in one study [21], while serum IgG titre against *A. ceylanicum* adult worm excretory–secretory proteins was assessed in another [23]. CRP and or AGP were quantified in only two independent articles [22,24].

For leishmaniasis, 14 biomarkers were quantified in total from the 5 independent studies. The most quantified micronutrients were zinc, copper and iron from three studies [31,33] with an additional fourth study where iron was also quantified [30]. Serum selenium concentration was measured in two studies [31,32], while serum albumin and ferritin were measured in two others [30,32]. Finally, growth hormone-insulin-like growth factor-1 was measured in a single independent study [30] as were calcium and magnesium [33]. Inflammation markers including interleukin (IL)-1β, IL-6, IL-8, tumour necrosis factor (TNF)-α and IL-2r were measured in a single study [32]. Four biomarkers relevant for serum nutritional status in leprosy are ferritin, vitamin D, vitamin A and cathelicidin, which were quantified in both qualifying studies [35,36].

### Meta-analysis

A total of 12 studies had similar methods and assessed the same biomarkers, which allowed for meta-analysis. Three types of meta-analyses were conducted, the first using five studies on intestinal parasites and its association with anthropometry, the second using three studies on leishmaniasis and its association with nutritional biomarkers and the third analysis conducted for intestinal parasites and leprosy and their association with nutritional deficiencies using two articles for each NTD.

### Association between intestinal parasites and anthropometry

**Fig. 2.**
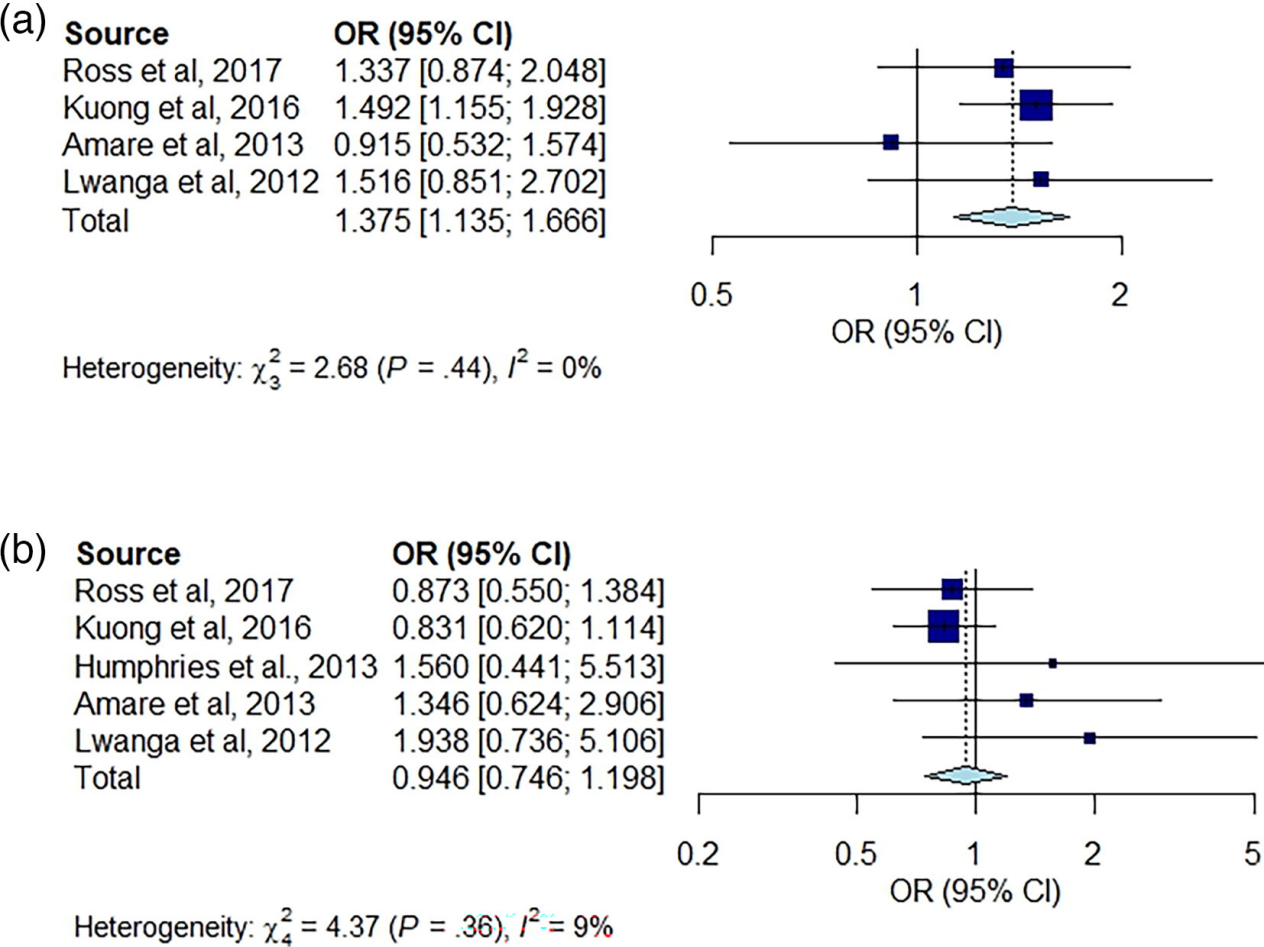
Forest plots for random effect meta-analysis of the association between the prevalence of (**a**) stunting (using the HAZ) and (**b**) Thinness/wasting (using BAZ) between those with and without intestinal parasites. The Mantel–Haenszel method was used to pool the OR, and 95% CIs were estimated using Clopper–Pearson’s method and REM likelihood estimator for tau^2^. The CIs of tau^2^ and tau were estimated using the Q-Profile method. The blue squares represent the absolute ORs, and the horizontal lines running through the blue lines represent the CI. The size of the boxes is equivalent to the weight of the study on the OR. Reference line (vertical middle line), overall effect (diamond) and direction of effect (dotted lines). We pooled the data across the studies that identified different intestinal parasites; Ross *et al.* [26] studied the infections of STH and schistosomiasis, Kuong *et al.* [24] looked for intestinal parasites (95% of which were hookworm infection), Humphries *et al.* [23] investigated only hookworm, Amare *et al.* [21] looked for intestinal parasites (discovering STH and a range of other organisms) and Lwanga *et al.* [25] looked for intestinal parasites (discovering STH and schistosomiasis).

The pooled association between BAZ and HAZ for intestinal parasites is shown in Fig. 2. Even though these studies identified a range of different intestinal parasites, the pooled estimate of association indicated that the prevalence of stunting (determined by HAZ) was significantly higher in children with intestinal parasites compared with uninfected children (OR = 1.38, 95% CI: 1.14–1.66, *I*^2^ = 0%, tau^2^ = 0). There was 0% heterogeneity across the studies as indicated by the *I*^2^ value. Among individual studies, only Amare *et al*, 2013 [21] reported no significant association between stunting and intestinal parasites (Fig. 2). On the other hand, the pooled estimate of the association between thinness/wasting and intestinal parasites was not statistically significant (OR = 0.95, 95% CI: 0.75–1.20, *I*^2^ = 9%, tau^2^ = 0.0036, Fig. 2b).

### Association between nutritional biomarkers and leishmaniasis

**Fig. 3.**
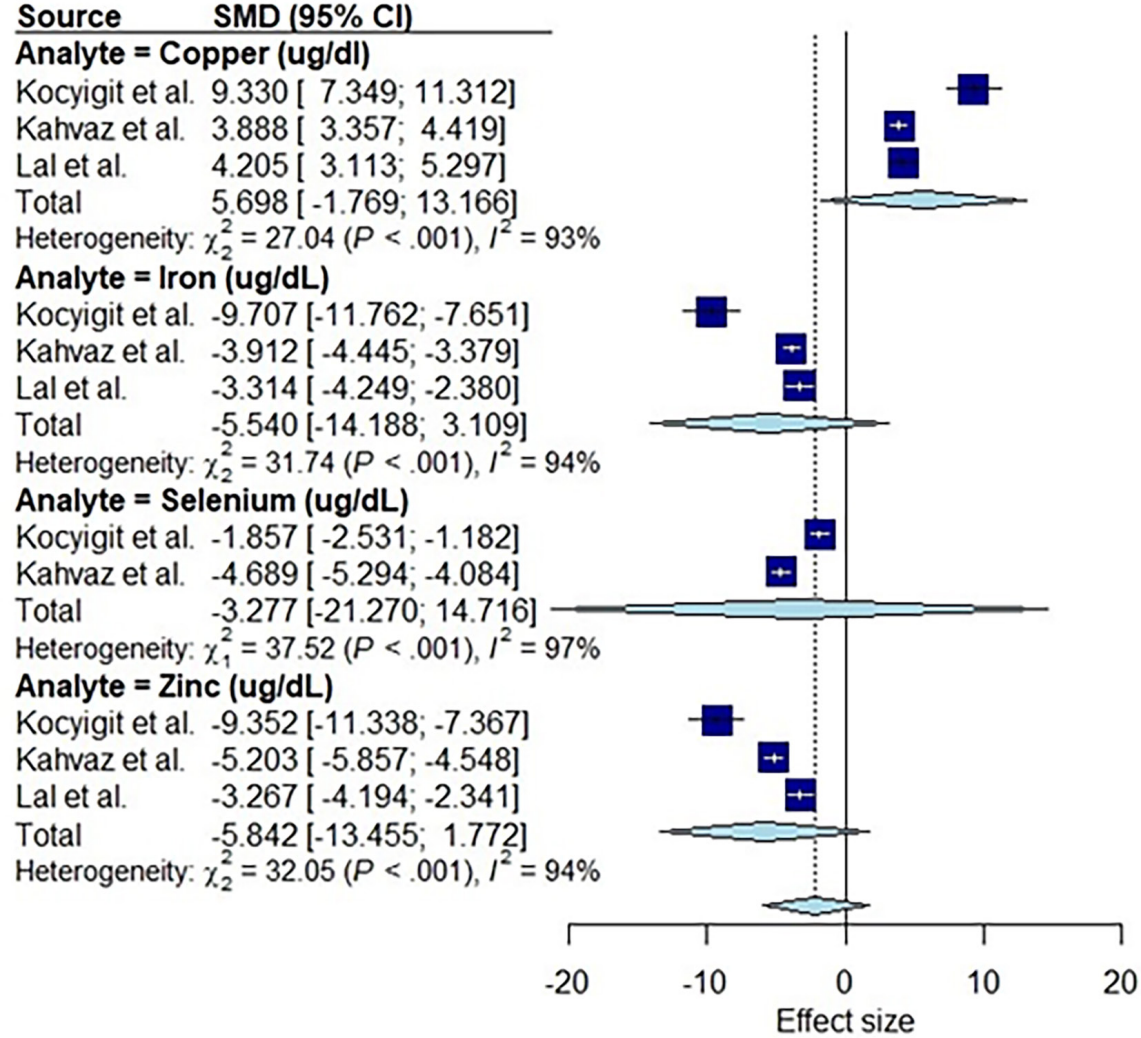
Forest plot showing overall pooled effect size estimate of leishmaniasis on nutritional serum biomarkers. The serum nutritional biomarkers that qualified for this analysis were copper, iron, selenium and zinc. The forest plot is presented as SMD and 95% CI. SMD in methods with sample correction was used to pool the mean differences between leishmaniasis cases and controls. The blue squares represent the SMD, and the horizontal black or white lines running through the squares represent the CI (white lines are used to visualize CIs that are smaller than the SMD). The size of the boxes is equivalent to the weight of the study on the pooled estimate. Reference line (vertical middle line), Hedges’ *g* (overall effect) is shown by the ‘diamond’; direction of effect (dotted lines). We pooled the data across the studies that studied different presentations of leishmaniasis; Kahvaz *et al.* [31] and Kocyigit *et al.* [32] studied cutaneous leishmaniasis, whereas Lal *et al.* [33] studied visceral leishmaniasis.

The individual studies reporting on the effect of Leishmania infection on nutritional markers were pooled in a sub-group analysis and shown in the forest plot (Fig. 3). Three studies that reported on the effect of leishmaniasis on copper levels (Fig. 3) showed consistent outcomes among the studies, i.e. an increase in copper levels in cases compared to controls. However, the pooled effect according to the random effect model is *g* = 5.70 (*g* = coefficient of means), with the 95% CI ranging from −1.77 to 13.17, and tau^2^= 19 881 was not statistically significant (*P* > 0.05). Similarly, three studies reported an apparent consistent decrease in iron and zinc in cases compared to controls; however, the pooled results were not statistically significant for zinc (*g* = −5.84, 95% CI: −13.45 to 1.77, *I*^2^ = 97%, tau^2^ = 201) or iron (*g* = −5.54, 95% CI: −14.19 to 3.11, tau^2^ = 253). Similar results were obtained for the two studies that measured selenium (*g* = −3.28, 95% CI: −21.27 to 14.72, tau^2^ = 4.21) with a consistent decrease in cases when compared to controls, which was also not statistically significant.

### Association between NTDs and nutritional deficiencies/insufficiencies

**Fig. 4.**
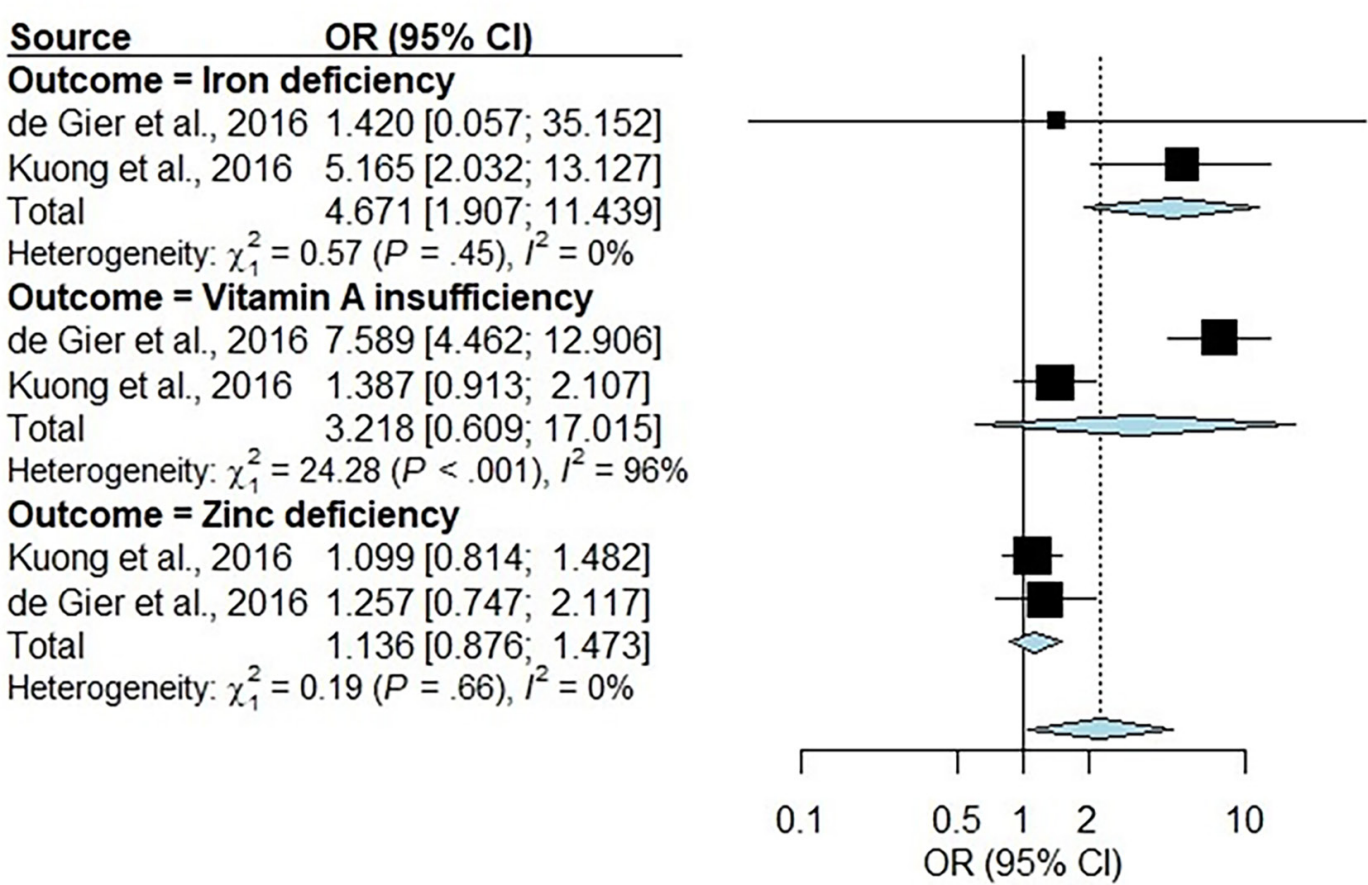
Sub-group analysis of an association between intestinal parasites and nutrient deficiencies/insufficiencies organized by nutrient type. The Mantel–Haenszel method was used to pool the OR, and 95% CIs were estimated using Clopper–Pearson’s method and REM likelihood estimator for tau^2^. The CIs of tau^2^ and tau were estimated using the Q-Profile method. The black squares represent the absolute ORs, and the horizontal lines running through the blue lines represent the CI. The size of the boxes is equivalent to the weight of the study on the OR. Reference line (vertical middle line), overall effect (diamond) and direction of effect (dotted lines). We pooled the data across the studies that identified different intestinal parasites; Kuong *et al.* [24] looked for intestinal parasites (95% of which were hookworm infection), whereas de Gier *et al.* [22] studied the infections of any STH (*A. lumbricoides*, *T. trichiura* or hookworm). The criteria cut-offs for the determination of iron deficiency, vitamin A insufficiency and zinc deficiency were similar across the two studies. Low ferritin (corrected value <15 µg l^−1^) was used as an indicator of depleted iron stores and high transferrin receptor (>8.3 mg l^−1^) as an indicator of iron tissue deficiency. Iron deficiency was defined using both ferritin and transferrin receptor indicators, that is, by depleted iron stores or iron tissue deficiency. Body iron was calculated from ferritin corrected for inflammation and transferrin receptor, as described by Cook *et al.* [64]. Serum retinol is bound to retinol-binding protein in a one-to-one complex; hence, retinol-binding protein concentrations were used to evaluate vitamin A (VA) status. Retinol-binding protein concentrations were adjusted for the presence of inflammation using correction factors of 1.15, 1.32 and 1.12 for incubation, early convalescence and late convalescence phases, respectively. Corrected retinol-binding protein cutoffs were used to define marginal VA status (<1.05 µmol l^−1^), vitamin A deficiency (VAD, <0.70 µmol l^−1^) and severe VAD (<0.35 µmol l^−1^), respectively. Zinc deficiency was defined using the following cutoffs: serum zinc <9.9 µmol l^−1^ for age 4–9 years, <10.1 µmol l^−1^ for girls ≥10 years of age and <10.7 µmol l^−1^ for boys ≥10 years of age. Severe zinc deficiency was defined as serum zinc <7.6 µmol l^−1^ [22,24].

Two studies [22,24] that reported the association between intestinal parasites and nutrient deficiencies of iron, vitamin A and zinc were pooled (Fig. 4). For zinc, the pooled OR was 1.14 (95% CI: 0.87–1.47, *I*^2^ = 0%, tau^2^ = 0), which was not statistically significant. Both vitamin A insufficiency and iron deficiency showed inconsistent results between the two studies included in the meta-analysis, but while vitamin A insufficiency did not reach statistical significance [3.22 (95% CI: 0.61–17.01, *I*^2^ = 96%, tau^2^ = 1.38), the pooled effect on iron deficiency was significant with no heterogeneity observed [OR = 4.67 (95% CI: 1.91–11.44, *I*^2^ = 0%, tau^2^ = 0)].

**Fig. 5.**
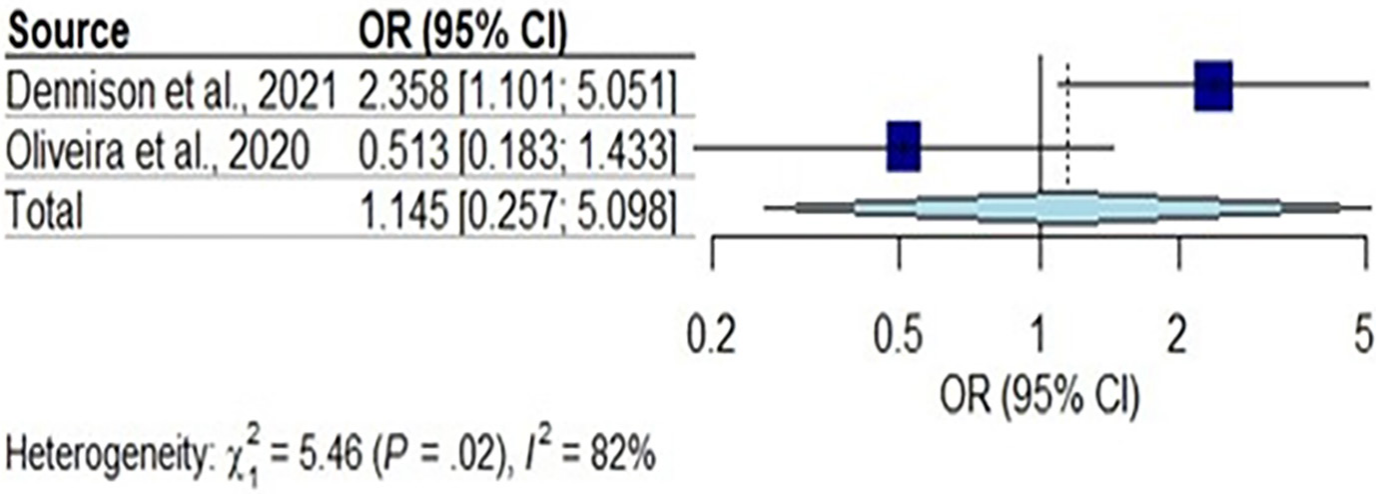
Sub-group analysis of an association between leprosy and vitamin D deficiency. The Mantel–Haenszel method was used to pool the ORs, and 95% CIs were estimated using Clopper–Pearson’s method and REM likelihood estimator for tau^2^. The CIs of tau^2^ and tau were estimated using the Q-Profile method. The blue squares represent the absolute ORs, and the horizontal lines running through the blue lines represent the CI. The size of the boxes is equivalent to the weight of the study on the OR. Reference line (vertical middle line), overall effect (diamond) and direction of effect (dotted lines). We pooled the data across the two studies that identified the association between vitamin D deficiency and leprosy; Dennison *et al.* [36] and Oliveira *et al.* [35]. Both studies used a cut-off for vitamin D deficiency of 25-OH vitamin D <20 µg l^−1^.

Two further studies considered an association between vitamin D deficiency and leprosy [35] and or leprosy/intestinal parasite coinfection [36] (Fig. 5**)**. The overall random pooled effects of the two studies 1.15 (95% CI: 0.26–5.10, *I*^2^ = 82%, tau^2^ = 0.951) did not reach statistical significance. There was 82% significant heterogeneity across the studies as indicated by the *I*^2^ value.

### Publication bias

**Fig. 6.**
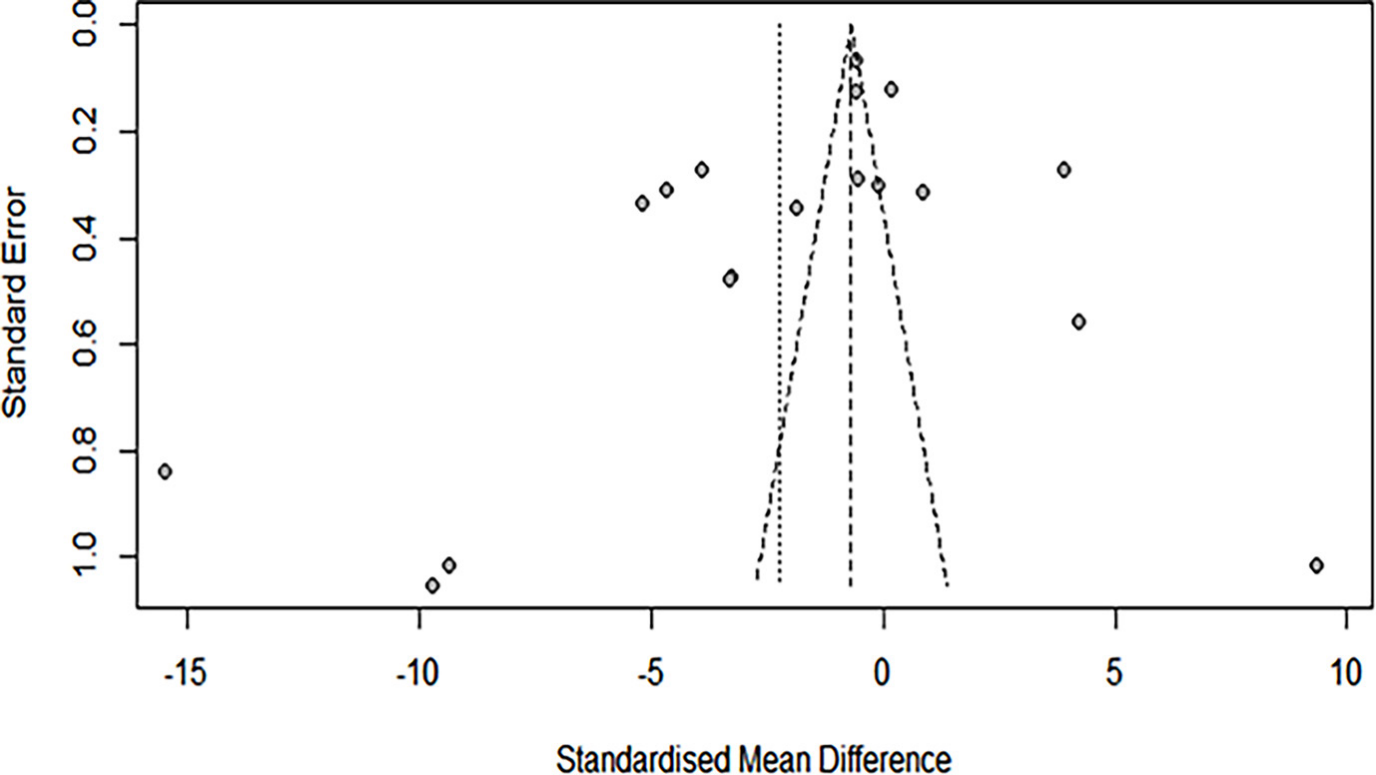
Funnel plot of SMD against standard error estimate of included studies. Individual studies are asymmetrically distributed along the vertical complete line. The scattered dots represent individual studies included in the meta-analysis. This shows that there is significant publication bias due to the asymmetrical distribution of effects in all included studies.

We found that there is a significant publication bias among the NTDs that qualified for the meta-analysis (Fig. 6). In addition, there was significant heterogeneity between the studies in all the sub-groups (asymmetry in the funnel plot, *Q* and *I*^2^ statistics). Meta-regression analysis was not performed because of the small study effect obtained with the sub-group analysis.

## Discussion

This systematic review and meta-analysis summarized primary research studies conducted globally that investigated the association between NTDs and malnutrition with all available data discovered and published in the past 2 decades (1999–2021). The 16 qualifying articles predominantly concerned intestinal parasites and were conducted in Asia. Studies from Africa were the least frequent out of the three continents. This geographic diversity may be one reason why food consumption assessments were difficult to analyse for the meta-analysis due to the variations in the foods across countries, nutritional content and approaches to estimation.

The NTDs with publications that qualified for this review and their association with nutrition and their inter-relation with immune response are discussed below.

### Intestinal parasites including STH and schistosomiasis

Intestinal parasites are an extremely well-studied group of parasitic nematode worms that have been reviewed in detail elsewhere [8]. Globally, around 24% of the world’s population are infected with an STH, and >250 million people need preventative treatment for schistosomiasis per annum. Importantly, STH impairs physical and mental development, especially in childhood, a finding corroborated by one study included in this review [24]. Helminths are known to have complex life cycles within the human host, which involves the subversion of the host immune system [38]. Classically, this involves mast cells, eosinophils and basophils and affects the Th1 and Th2 balance that may also explain the relationship identified between allergy, anthropometric values and intestinal parasite infection in one qualifying study [21].

The nutritional associations and impact of helminth infection are recognized by the WHO and have been reviewed elsewhere [39]. In particular, intestinal parasites cause iron deficiency anaemia and reduce the growth rate of children that can, in some severe cases, be fatal. Indeed, our meta-analysis corroborated the association between anthropometry and intestinal parasites. On the other hand, a 2014 meta-analysis of 37 observational and randomized controlled trials in school-aged children found a link between helminth infection and serum retinol, but not serum ferritin [40]. While we were unable to perform meta-analysis for iron levels, some studies did report reduced blood iron, likely due to the loss of blood as a typical occurrence in the host during worm infestation [39].

### Leishmaniasis

Leishmaniasis is caused by a protozoan parasite that is transmitted by sandflies, reviewed in [41]. About 0.7–1 million new cases are reported annually with 90% of these reported in low- and middle-income countries. As an intracellular parasite that infects innate immune cells, the immune response is crucial to outcomes [42]. However, in papers qualifying for this review, a single study quantified immune markers [32]. Here, the inflammation markers IL-1β, IL-6, IL-8 and TNF-α were elevated in the cases compared to the controls. This is in line with the recent transcriptomic data showing that an inflammatory signature is present in circulating leukocytes [43].

For leishmaniasis, meta-analysis was only possible for the assessment of nutritional biomarkers. However, none of these three reports corrected biomarkers for systemic inflammation by measurement of CRP and/or AGP (see below). Copper levels were found to be consistently elevated in cases compared to controls, whereas selenium, zinc and iron levels were lower [31,33]; however, none of these reached statistical significance in our meta-analysis. Others have also compared serum zinc levels between acute and chronic forms of leishmaniasis patients in Iran. The zinc levels were found to be significantly lower in cases (acute at 50% and chronic at 43.3%) compared to controls (13.3%). The impaired levels of micronutrients in the serum were attributed to nutritional deficiencies and underlying diseases [44]. A previous systematic review of 29 articles on trace element status in human and animal leishmaniasis also independently made similar conclusions [45]. They also found a significant decrease in the level of iron and zinc in both human cutaneous and visceral leishmaniasis and, like us, found that copper was increased [45]. This suggests that susceptibility to leishmaniasis may be associated with alterations in the levels of trace elements and that correcting these may be of clinical benefit.

### Leprosy

Leprosy is a mycobacterial disease of the skin, peripheral nerves and mucosa of the upper respiratory tract, reviewed in [46]. With more than 200, 000 cases reported each year, it remains an important problem particularly in Brazil, India and Indonesia [47]. *M. leprae* is an obligate parasite of macrophages, and the type of leprosy reaction is dependent on the immune response invoked [46].

In the current review, a sub-group meta-analysis from two case–control studies was only possible for the prevalence of vitamin D deficiency, and this did not reach statistical significance. Even though the vitamin D deficiency pooled effects from the two studies were not statistically significant, the leprosy cases had higher proportions of vitamin D deficiency compared to the control groups. In a recent narrative review, cases were reported to have lower levels of vitamin D [48]. This nutritional deficiency may be attributed to the vitamin D receptor (VDR), which is essential for immunomodulatory activity by regulating antigen-presenting cells and T-cell function [49]. Vitamin D has also been shown to induce the production of antimicrobial peptides (cathelicidins and defensins) by infected macrophages that kill mycobacteria [50,51]. This association was demonstrated in a study, which showed that VDR gene expression was lower in leprosy patients compared to controls [52].

### Way forward

One of the single most relevant and recent advances in nutrition research relating to infectious diseases is that we now know that systemic inflammation can lead to misdiagnosis of plasma micronutrient deficiency. Hence, plasma CRP can categorize minor (<10 mg l^−1^), moderate (11–80 mg l^−1^) and major (>80 mg l^−1^) inflammation, which progressively lowers plasma levels of micronutrients (zinc; selenium; vitamins A, D, E, K, B2, B6, B12 and C; lutein; lycopene; and *α*- and *β*-carotene) [53]. Independently, it was found that, during major inflammation (CRP >80 mg l^−1^), vitamins A and B6 fell by 40%, while vitamin C and carotenoids lutein, lycopene and *α*- and *β*-carotene fell by 80% [54]. Taken together, it is now clear that CRP and/or AGP should be quantified when measuring these micronutrients, so that any changes in micronutrient levels in diseases driven by chronic inflammation can be adjusted. It is worth noting that not all NTDs are associated with systemic inflammation, with Buruli ulcer and STH [55,56].

While adjustment protocols for some measures related to anaemia have been developed [57], which have recently been extended to include zinc and vitamins B12 and D [58], concerns remain about introducing additional variability by using correction factors that do not always correlate. Nevertheless, it is likely to be appropriate to use this approach in the future for the three disease areas reviewed here because all have been shown to be associated with systemic inflammation. In leishmaniasis, there are increased levels of proinflammatory mediators including TNF-α, CRP and adenosine deaminase [59]. Furthermore, there is good evidence for systemic inflammation in visceral leishmaniasis from clinical studies that used acute phase proteins as predictive markers for parasite clearance; here, patients were found to present with elevated levels of CRP, serum amyloid A protein and AGP at baseline, which decreased after 3 months of therapy [60,61]. Leprosy disease pathogenesis also shows local inflammation, which later becomes systemic leading to elevated concentration of proinflammatory markers such as CRP, IL-6, and interferon-γ [46]. Moreover, the populations at risk of NTDs often have a high baseline of inflammation even in the absence of these diseases [56], which may also be caused by co-infection with other organisms which then leads to a syndrome known as environmental enteric dysfunction (EED) [62].

Going forward, researchers should therefore quantify serum CRP and/or AGP levels to correct micronutrient levels for systemic inflammation if they are above recommended cut-offs [58]. It is critical to note that the aforementioned diseases have phases of disease progression, indicating that if systemic inflammation is not confirmed at baseline, there will be no need to adjust for micronutrient levels. In leprosy, for example, the disease progression takes as long as 7 years, which may be responsible for the difficulty in determining systemic inflammation. It may also be wise to assess whether there is EED in the participants recruited to such studies.

In addition to greater consideration of systemic inflammation, more mechanistic investigations are needed to better understand the synergy between nutrition, infection and immunity introduced by Scrimshaw *et al.* [14]. A realistic pathway to increasing the knowledge of these interactions for under-funded or the more-neglected NTDs is the integration agenda, driven by the WHO. A good example here is their current focus on skin NTDs, where a framework to evolve prevention, diagnosis and treatment is being developed [63]. As many people infected with one NTD may also have another, understanding the potential interactions between these different infections in terms of susceptibility, immune response and impact on micronutrients will be of relevance for future studies.

### Strengths and limitations

The main strength of this systematic review and meta-analysis is that for the first time, we take an overarching view of the current state of research into nutrition and NTDs. This identified an association between micronutrient inadequacy and intestinal parasites, leprosy and leishmaniasis. We also identified the main biomarkers that seem to be key in serum nutritional assessments in intestinal parasites, leishmaniasis and leprosy. However, it also identified major gaps in knowledge.

We prioritized our review of articles that aimed to evaluate the association between NTD diagnosis vs. controls and malnutrition. The study was limited by the heterogeneity of publications between the four NTDs. Intestinal parasites (STH and schistosomiasis) were more frequently studied than other diseases. This bias affected the outcomes from the meta-analysis but is a true reflection of the focus of research in these diseases globally. Our review and meta-analysis focussed on case–control studies. A major weakness therefore is that we excluded many studies that could have been informative because of the study design. Studies that did not have a control group but focussed only on different clinical forms of an NTD or a group of NTDs were also not included, neither were clinical trials nor intervention studies.

Finally, there are limitations relating to our search strategy. First, our search approach focusing on disease names may have missed some relevant articles that would have been found if we expanded our search terms to include NTD infectious organisms. Second, one of the inclusion criteria was articles written in English only. This meant that literature written in other languages was excluded. We acknowledge particularly that French and Arabic are the official languages in 21 and 12, respectively, of the 54 African countries (nearly 60% of total). This may have limited the number of studies from Africa that could be included in this review.

## Conclusion

In conclusion, this review identified associations between infections, nutrition and anthropometric biomarkers for only three NTDs: intestinal parasites, leprosy and leishmaniasis. Moreover, even though the burden of NTDs in African countries is high, few studies took place here (even for intensely studied intestinal parasites). Therefore:

- Research on NTDs needs to be prioritized in the African countries where their burden is high.
- Research on NTDs, other than intestinal parasites, should be increased.
- The role of diet and the correlation of poverty in the pathogenesis of skin NTDs such as Buruli ulcer, yaws and scabies should be further investigated.
- Studies to establish the nutritional/dietary status of people infected with such diseases need to be funded.

However, the impact of systemic inflammation on micronutrients must be taken into account when interpreting this data. Selected micronutrient deficiencies seem to be defining markers to unravel whether the disease susceptibility results from prevailing nutritional deficiency, or whether these nutritional deficiencies are caused by the infection. The nutritional status prevailing in NTD-endemic communities, as well as prospective studies, will give more clues on their association with disease, including susceptibility, response to treatment and re-infection risk.

## supplementary material

10.1099/acmi.0.000800.v3 Uncited Supplementary Material 1.

## Abbreviations

AGP: acid glycoprotein
BAZ: body mass index-for-age
BMI: body mass index
CI: confidence interval
CRP: C-reactive protein
EED: environmental enteric dysfunction
FFQ: food frequency questionnaire
HA: height-for-age
HAZ: HA Z score
IL: interleukin
MUAC: mid-upper arm circumference
NOS: Newcastle–Ottawa scale
NTDs: neglected tropical diseases
OR: odds ratio
PRISMA: Preferred Reporting Items for Systematic Review and Meta-Analysis
REM: restricted maximum
SMD: standardized mean difference
STH: soil-transmitted helminthiasis
TNF-α: tumour necrosis factor alpha
VDR: vitamin D receptor
