# Supplementary material for: A systematic review and meta-analysis of the association between neglected tropical diseases and malnutrition: more research needed on diseases other than intestinal parasites, leishmaniasis and leprosy
Source: Access Microbiol. 2024 Nov 13;6(11):000800.v3. doi: 10.1099/acmi.0.000800.v3 (PMC11559247; doi:10.1099/acmi.0.000800.v3)
Supplement: Uncited Supplementary Material 1. [file acmi-6-00800-s001.pdf]

## *Supplementary Material*

### 1 Supplementary Data

**Supplementary Table 1:** Search strategy

| **Key concepts** | **Exposure variables** | **Outcome variables** | **Exposure variables versus outcome variables** |
|---|---|---|---|
| **Search terms** | "Nutritional status" OR<br><br>"macronutrients" OR<br><br>"micronutrients" OR<br><br>"anthropometrics" OR<br><br>"dietary intake" | "NTD" OR<br><br>"Neglected tropical diseases" OR<br><br>"Ascariasis" OR<br><br>"Trichuriasis" OR<br><br>"Hookworm infection" OR<br><br>"Schistosomiasis" OR<br><br>"lymphatic filariasis" OR<br><br>"Trachoma" OR<br><br>"Onchocerciasis" OR<br><br>"Leishmaniasis" OR<br><br>"Chagas disease" OR<br><br>"Leprosy" OR<br><br>"Human African trypanosomiasis" OR<br><br>"Dracunculiasis" OR | "Nutritional status" OR<br><br>"macronutrients" OR<br><br>"micronutrients" OR<br><br>"anthropometrics" OR<br><br>"dietary intake"<br><br>**AND**<br><br>"Neglected tropical diseases" OR<br><br>"Ascariasis" OR<br><br>"Trichuriasis" OR<br><br>"Hookworm infection" OR<br><br>"Schistosomiasis" OR<br><br>"lymphatic filariasis" OR |

| | | | |
|---|---|---|---|
| | | "Buruli ulcer" OR<br><br>"Yaws" OR<br><br>"Scabies" | "Trachoma" OR<br><br>"Onchocerciasis" OR<br><br>"Leishmaniasis" OR<br><br>"Chagas disease" OR<br><br>"Leprosy" OR<br><br>"Human African trypanosomiasis" OR<br><br>"Dracunculiasis" OR<br><br>"Buruli ulcer" OR<br><br>"Yaws" OR<br><br>"Scabies" |

**Table Supplementary Table 2:** Quality assessment results of articles included in this review.

| | Selection | | | | | Comparability | | Exposure | | | Quality scores | | |
|---|---|---|---|---|---|---|---|---|---|---|---|---|---|
| **Study/year** | **Is the case definition adequate?** | **Representativeness of the cases** | **Selection of Controls** | **Definition of Controls** | | **Comparability of cases and controls on the basis of the design or analysis** | | **Ascertainment of exposure** | **Same method of ascertainment for cases and controls** | **Non-Response rate** | | | **Reference** |
| Atukorala and Lanerolle, (1999) | * | * | * | 0 | | ** | | * | * | 0 | 7 | Good | (54) |
| Amare *et al*, (2013) | * | * | * | * | | * | | * | * | * | 8 | Good | (43) |
| de Gier *et al, (2016)* | * | * | * | * | | * | | * | * | * | 8 | Good | (52) |
| Humphries *et al, (2013)* | * | * | * | * | | * | | * | * | 0 | 7 | Good | (44) |
| Kuong *et al, (2016)* | * | * | * | * | | * | | * | * | 0 | 7 | Good | (45) |
| Lwanga *et al, (2012)* | * | * | * | * | | * | | * | * | * | 8 | Good | (46) |
| Quihui-Cota *et al*, (2010) | * | * | * | * | | * | | * | * | * | 8 | Good | (55) |
| Ross *et al, (2017)* | * | * | * | * | | ** | | * | * | * | 9 | Good | (47) |
| Zavala *et al, (2017)* | * | * | * | * | | * | | * | * | * | 8 | Good | (53) |
| Gomes *et al*, (2007) | * | * | * | * | | * | | * | * | * | 8 | Good | (56) |
| Goyonlo *et al, (2018)* | * | * | * | * | | * | | 0 | 0 | * | 6 | Good | (60) |
| Kahvaz *et al*, (2020) | * | * | * | * | | * | | * | 0 | * | 7 | Good | (57) |
| Kocyigit *et al*, (2002) | * | * | * | * | | * | | * | 0 | * | 7 | Good | (58) |
| Lal *et al, (2013)* | * | 0 | 0 | * | | * | | * | * | * | 6 | Good | (59) |
| Dennison *et al*, (2021) | * | * | * | * | | ** | | * | 0 | 0 | 7 | Good | (62) |
| de Oliveira *et al* (2020) | * | * | * | * | | ** | | * | 0 | * | 8 | Good | (61) |
| *Wagenaar et al, (2015)* | * | 0 | * | * | | * | | 0 | 0 | 0 | 4 | Poor | |
| *de Castilhos et al, (2017)* | * | * | 0 | 0 | | * | | * | 0 | 0 | 4 | Poor | |
| Oktaria et al, (2018) | 0 | 0 | * | 0 | | * | | 0 | 0 | * | 3 | Poor | |
| *Fairley et al, ( 2019)* | * | * | 0 | 0 | | * | | 0 | * | 0 | 4 | Poor | |

Good quality: 3 or 4 stars in the selection domain AND 1 or 2 stars in the comparability domain AND 2 or 3 stars in the outcome/exposure domain. Fair quality: 2 stars in the selection domain AND 1 or 2 stars in the comparability domain AND 2 or 3 stars in the outcome/exposure domain. Poor quality: 0 or 1 star in selection domain OR 0 stars in comparability domain OR 0 or 1 stars in outcome/exposure

domain. The four articles that did not pass the NOS test (Poor quality score) and was not included in the systematic review were highlighted with an orange colour. They are Wagenaar *et al*, (2015), de Costilhos *et al*, (2017), Oktaria *et al*, (2018), Fairley *et al*, (2019).
